# An Exploratory Study of Cannabidiol as an Adjunctive Treatment for Refractory Epilepsy in Dogs

**DOI:** 10.3390/ani15243614

**Published:** 2025-12-15

**Authors:** Kanogwan Kimram, Nirut Suwanna, Bordin Tiraphut, Sasithorn Limsuwan, Suporn Thongyuan, Natthasit Tansakul

**Affiliations:** 1 Graduate Program in Animal Health and Biomedical Sciences, Faculty of Veterinary Medicine, Kasetsart University, Bangkok 10900, Thailand; drkimgolf@gmail.com; 2 Veterinary Teaching Hospital, Faculty of Veterinary Medicine, Kasetsart University, Bangkok 10900, Thailand; fvetbdt@hotmail.com; 3 Department of Companion Animal Clinical Sciences, Faculty of Veterinary Medicine, Kasetsart University, Bangkok 10900, Thailand; nirut.s@ku.th; 4 Institute of Food Research and Product Development, Kasetsart University, Bangkok 10900, Thailand; sasithorn.limsu@ku.th; 5 Department of Veterinary Public Health, Faculty of Veterinary Medicine, Kasetsart University, Nakhon Pathom 73140, Thailand; fvetspty@ku.ac.th; 6 Department of Pharmacology, Faculty of Veterinary Medicine, Kasetsart University, Bangkok 10900, Thailand

**Keywords:** cannabidiol, CBD, epilepsy, dog, quality of life

## Abstract

Canine epilepsy often resists conventional antiepileptic drugs (AEDs), which affects their quality of life. Cannabidiol (CBD) has anticonvulsant properties; however, evidence of its use in canine epilepsy is limited and contradictory. This prospective pilot study aimed to investigate the potential advantages, safety profile, and effects of CBD on quality of life when used as an adjunctive therapy in cases of drug-resistant epilepsy in canines. Thirteen dogs with refractory epilepsy, all on 2–6 concurrent AEDs, were enrolled. A single-arm pretest–post-test design was used. CBD was titrated from 0.5 mg/kg BID 2.5 mg/kg q12h. The primary outcome was the change in seizure frequency. Secondary outcomes included changes in seizure severity, seizure cluster, hematological and biochemical parameters, and owner-reported quality of life (QoL). Significant overall seizure frequency reduction (*p* = 0.02) with the median decreased from 11 (IQR 9–22) during the pre-intervention period to 5 (IQR 2–13) at the post-intervention follow-up. Notably, 61.5% of the dogs achieved a ≥50% reduction in seizure frequency. The number of seizure clusters was significantly decreased (*p* = 0.001). Most hematological/renal parameters remained stable; however, Alkaline Phosphatase (ALP) levels significantly increased (*p* < 0.001). The owners reported positive CBD perceptions and an improved quality of life. CBD shows the potential for refractory canine epilepsy, especially in clusters. Increased hepatic enzyme levels necessitate rigorous monitoring, particularly with the concurrent use of AEDs. This groundbreaking study explored the application of CBD in managing canine epilepsy, utilizing a “start-low, go-slow” strategy to minimize adverse effects while effectively controlling seizures. Our findings underscore the necessity of customizing CBD dosages for individual needs and highlight the critical importance of monitoring liver function. This study challenged the traditional one-size-fits-all dosing approach. It provides the first evidence and practical framework for the use of CBD to treat canine epilepsy in Asia, detailing the pioneering approach and the initial findings from this cohort.

## Simple Summary

Canine epilepsy often resists conventional antiepileptic drugs (AEDs), affecting quality of life. Cannabidiol (CBD) has anticonvulsant properties; however, evidence of its use in canine epilepsy is limited and contradictory. This pilot study evaluated CBD’s potential efficacy, safety, and impact as an adjunct therapy for drug-resistant canine epilepsy. Thirteen dogs with refractory epilepsy, all on 2–6 concurrent AEDs, were enrolled. A 12-week, single-arm, pretest–post-test design was used. CBD was titrated from 0.5 mg/kg BID 2.5 mg/kg q12h. The key outcomes included seizure frequency/severity, cluster reduction, hematological/biochemical parameters, owner-reported adverse events, and quality of life. In the entire cohort, there was a notable reduction in the overall seizure frequency, with 61.5% of the individuals experiencing a reduction of 50% or greater. Furthermore, a significant decrease in the number of seizure clusters was observed. Most hematological/renal parameters remained stable; however, Alkaline Phosphatase (ALP) levels significantly increased. The owners reported positive CBD perceptions and an improved quality of life. CBD shows the potential for refractory canine epilepsy, especially in clusters. Increased hepatic enzyme levels necessitate rigorous monitoring, particularly with the concurrent use of AEDs. A pragmatic “start-low, go-slow” titration strategy was used to optimize the safety and efficacy. These results provide novel clinical insights, indicating that effective dosing is highly personalized and requires a customized approach. To our knowledge, this is the first report of such a strategy and findings in an Asian canine population.

## 1. Introduction

Epilepsy in dogs is a complex neurological disorder characterized by recurrent spontaneous seizures of diverse etiologies [1,2]. Clinically, seizures range from mild focal episodes to severe generalized convulsions, and the condition is broadly classified as idiopathic epilepsy (IE) or structural epilepsy (SE), with IE being the most common form [3]. Canine epilepsy poses significant challenges in veterinary medicine, often necessitating lifelong anti-epileptic drug (AED) therapy, and profoundly impacts the quality of life of affected dogs and their owners [4,5]. Notably, the shared pathophysiological and clinical features between canine and human epilepsy make dogs a valuable translational model for studying disease mechanisms, treatment efficacy, and comorbidities [2,5].

Despite its clinical importance, the prevalence of canine epilepsy remains uncertain owing to inconsistencies in diagnostic criteria and study populations. Estimates range from 0.5% to 0.75% in the general dog populations [6]. Although conventional AEDs such as phenobarbital and potassium bromide are effective in many cases [1], 20–30% of dogs exhibit drug-resistant epilepsy [5]. Moreover, AED-related adverse effects further complicated management, underscoring the need for alternative therapies that balance seizure control with quality of life [4].

The endocannabinoid (EC) system has emerged as a potential modulator of neuronal hyperexcitability in epilepsy [7]. Cannabidiol (CBD), a non-psychoactive phytocannabinoid, has shown anticonvulsant properties in preclinical studies (e.g., mechanism of action and effects at the cellular level) and clinical studies (e.g., Lennox–Gastaut syndrome) [8,9,10,11], prompting interest in its use in refractory canine epilepsy. However, current evidence is limited and contradictory. While some studies have reported reduced seizure frequency [12], others have found no significant benefit [13,14]. These disparities may reflect variations in study design, CBD formulations, or population characteristics (e.g., epilepsy type and concurrent medications).

Given the translational relevance of canine epilepsy and the urgent need for effective therapies, this prospective pilot study aimed to evaluate the potential benefits and safety of CBD as an adjunctive treatment for dogs with drug-resistant epilepsy. This study focuses on three pivotal areas: seizure management, safety assurance, and quality-of-life enhancement. This investigation compared baseline and post-intervention outcomes, with the design selected to address the challenges of recruiting treatment-refractory cases and ensuring owner compliance in a practical clinical environment. By assessing CBD’s therapeutic potential in this understudied population, this study aimed to provide preliminary data for future larger-scale controlled trials and to address gaps in dosing protocols and variability in individual responses.

## 2. Materials and Methods

### 2.1. Ethics

This study was conducted with the approval of the Animal Care and Use for Scientific Research Committee at the Faculty of Veterinary Medicine, Kasetsart University, Bangkok, Thailand (ACKU 65-VET-061). The requirement for human ethical approval was waived (COE68/047). All the experimental methodologies and informed consent procedures were implemented in compliance with the institution’s core requirements.

### 2.2. Animals: Study Population and Eligibility Criteria

Of the 81 neurology appointments at Kasetsart University Veterinary Teaching Hospital, 35 were evaluated for suspected refractory epilepsy. Of these, 18 met the preliminary criteria, and 13 dogs were ultimately enrolled after the owner provided informed consent. Key inclusion criteria were diagnosis of refractory epilepsy, defined as failure to achieve sustained seizure freedom despite continuous treatment with at least two appropriately dosed, well-tolerated Anti-epileptic Drugs (AEDs). To qualify, dogs were required to have been on constant AED treatment for their epilepsy for over six months before study initiation and have been treated with their current number of AEDs for a minimum of three months. The minimum average seizure frequency was at least one seizure per month over the three months before enrollment. During the preceding three months, dogs should have remained on their existing medications, with no new AEDs introduced during this timeframe. A priori power analysis was conducted using G*Power 3.1.9.4 to determine the required sample size for the repeated measures within-factor design [14,15]. Given the effect size of f = 0.8, α = 0.05, and power = 0.95, the minimum required sample size is 12.

In relation to the diagnostic classification and pragmatic approach, the study protocol was adapted early in the design phase to address the logistical and financial challenges associated with completing the full International Veterinary Epilepsy Task Force (IVETF) Level II diagnostic workup for all participants in a clinical trial setting [16]. A pragmatic inclusion strategy was adopted to ensure enrollment of a statistically powered cohort. This strategy allowed the inclusion of dogs that met all critical clinical criteria for refractory epilepsy, such as documentation of two failed AEDs and exclusion of major systemic causes, even if they lacked some aspects of the full diagnostic workup (e.g., bile acid testing). The final study population consisted of 13 dogs: nine with Idiopathic Epilepsy (IE) (Tier Level I, n = 4; Tier Level II, n = 5) and four with Structural Epilepsy (SE). Dogs in the SE cohort were included only if Magnetic Resonance Imaging (MRI) revealed a static, non-progressive lesion (e.g., porencephaly) considered the epileptogenic focus, and Cerebrospinal Fluid (CSF) analysis excluded active, progressive Central Nervous System (CNS) disease.

All enrolled dogs received phenobarbital within the therapeutic range and at least one adjunctive AED (potassium bromide, gabapentin, levetiracetam, zonisamide, topiramate, or imepitoin). Stable non-neurological comorbidities and associated medications, including corticosteroid therapy (maintained at a consistent dosage for ≥three months), were permitted. During the cohort period, adjustments to existing medications for CBD treatment were allowed to manage the adverse effects, thereby ensuring adherence to the established protocol. The Supplementary Table S1 provides detailed information on demographic and medication data.

### 2.3. Study Design and Procedures

*Study Design and Primary Outcome*

This prospective clinical trial utilized a single-arm, pretest–post-test design to evaluate the therapeutic potential of CBD in canine refractory epilepsy. This study focused on three key domains: seizure control, safety profile, and QoL. Using a within-subjects framework, each dog’s performance during the 12-week treatment period was compared with its baseline during the 12-week pre-intervention phase. Seizure frequency and cluster occurrence were tracked across three consecutive 4-week intervals during both baseline and treatment periods. The primary efficacy outcome was the responder rate, defined as the proportion of dogs achieving ≥50% reduction in the mean monthly seizure frequency from baseline to the treatment period.

*Data Collection and Secondary Assessments*

At the initial consultation, pet owners were provided with a seizure diary to record all seizure events, potential cluster activity, and CBD dosing throughout the study. Seizure frequency was defined as the total number of seizures occurring within a standardized 4-week interval. A seizure cluster was defined as two or more seizures occurring within a 24-h period. The total number of seizure events was calculated from the diaries, with each seizure within a cluster counted as a distinct event. Seizure events were evaluated in each phase of the study (12-week baseline and 12-week treatment periods, each comprising three 4-week intervals).

Secondary outcomes, including reductions in seizure frequency and clusters, were assessed during the monthly comprehensive evaluations. Safety was closely monitored via therapeutic drug monitoring to screen for CBD-AED interactions and serial hematological and biochemical analyses.

Upon completion of the study, the owners completed the final survey (details are provided in Supplementary Table S2). This survey used a five-point Likert scale to evaluate the incidence of 11 predefined adverse events or behavioral changes (e.g., loss of balance, vomiting, diarrhea, increased appetite, restlessness, and hypersalivation). The survey also collected final owner-reported data on changes in dogs’ quality of life, treatment response (including seizure severity), and overall attitudes towards CBD use.

*Dosing Strategy*

In contrast to previous protocols that implemented a fixed, high-dose CBD regimen (2.5 mg/kg q12h), we elected to use a modified ‘start-low, go-slow’ titration approach. This decision was driven by preliminary safety findings from an earlier distinct cohort (unpublished data), which revealed dose-limiting adverse effects and raised owners’ concerns about starting at the maximum dose. At the beginning of the study, all the dogs received a CBD dosage of 0.5 mg/kg q12h. The potential for weekly dose escalation was evaluated based on the absence of significant adverse effects, insufficient seizure control, and owner approval. The dosage could be increased by 0.25–0.5 mg/kg through a weekly consultation, with a maximum dose of 2.5 mg/kg q12h. The final dosing decision was made jointly by the owner and clinical investigator based on the balance between seizure control and observed adverse effects.

### 2.4. CBD Product Preparation

The CBD product was modified and analyzed as described previously [17,18]. Certified CBD powder with purity exceeding 99%, as reported by a certified third-party test laboratory, was obtained from Salus Bioceutical (Thailand) Co., Ltd., Bangkok, Thailand. An oil-based CBD preparation method has also been developed. Isolated CBD powder (15 g) was weighed into a volumetric flask and dissolved in 300 mL of oil. The mixture was then dispersed using a magnetic stirrer on a warm plate at approximately 45 °C for 30 min. The HPLC-DAD (Thermo Scientific^TM^ Vanquish^TM^ Core HPLC systems, Waltham, MA, USA) in-house validation method for CBD quantification demonstrated linearity over the concentration range 0.01–0.4 mg/L. This method yielded a coefficient of determination ≥ 0.999 and achieved an LLOQ of 0.01 mg/L. The precision and accuracy of the method were determined to be within 3.60–4.18% and 95.6–102.4%, respectively. The concentrations of all CBD products were verified using assays conducted both before and after canine administration (monthly sampling).

### 2.5. Statistical Analysis

Statistical analyses were conducted using Stata statistical software Release 19 (StataCorp LLC, College Station, TX, USA). GraphPad Prism version 9 (GraphPad Software, LLC, Boston, MA, USA) and R version 4.3.3 [19]. Descriptive statistics, including frequency, mean, standard deviation (SD), median, and interquartile range (IQR), were used to summarize the principal characteristics of the study. The normality of the data was evaluated using the Shapiro–Wilk test, while homogeneity of variance was assessed using Mauchly’s test of sphericity. To evaluate the adverse effects of CBD, one-way repeated-measures ANOVA and post hoc multiple-comparison tests, in conjunction with the Friedman test, were used to examine differences in 18 blood parameters between the baseline (12-week period) and post-treatment (4, 8, and 12 weeks) periods. To assess the effect of CBD on seizure frequency and seizure clusters, a Wilcoxon matched-pairs signed-rank test was conducted to compare pre- and post-treatment (12-week period) data. Furthermore, the Friedman test, followed by a Bonferroni correction for multiple comparisons, was used to assess differences in seizure frequency and seizure clusters between the baseline (12-week period) and post-treatment (4, 8, and 12 weeks) periods. Statistical significance was set at *p* < 0.05.

## 3. Results

### 3.1. Demographics and Clinical Characteristics

The study cohort comprised 13 dogs diagnosed with refractory epilepsy. The demographic data revealed a diverse patient population. Among the 13 participants, 10 were male, and three were female. The breeds represented varied, although small breeds were notably prevalent, including six Chihuahuas, one Pomeranian, one Labrador Retriever, three Siberian Huskies, one Beagle, and one crossbreed. The dogs’ ages at the commencement of the study ranged from 1.5 to 10 years, with seizure onset occurring from 5 months to 8 years earlier. The final cohort comprised nine patients with IE and four with SE. All 13 dogs were included in this study.

A defining characteristic of this study’s cohort, confirming the refractory nature of their epilepsy, was the administration of multiple concurrent AEDs for all 13 dogs, each receiving between 2 and 6 different medications. The most frequently administered AEDs were Phenobarbital, Zonisamide, Levetiracetam, and Potassium bromide (KBr). This polytherapy regimen underscores the severity and intractability of seizures before CBD intervention. Demographic and medication data are presented in the Supplementary Table S1.

### 3.2. Seizure Control Outcomes

*Seizure Frequency Outcomes*

**Figure 1:**
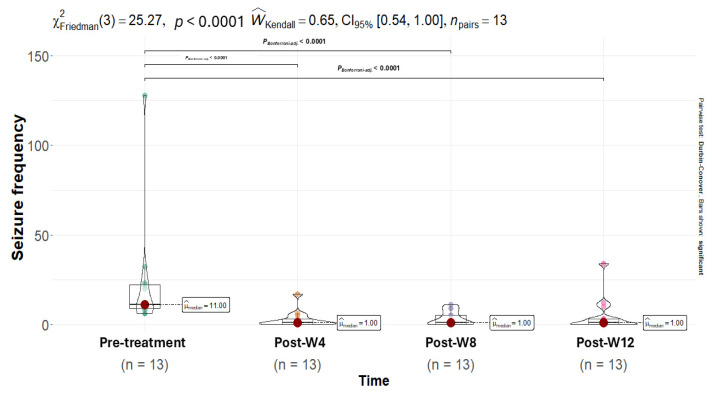
A Friedman test was conducted to compare seizure frequency across time points (pre- and post-intervention). The test indicated a significant difference in seizure frequency (χ^2^ = 25.27, *p* < 0.0001). Post hoc tests with the Bonferroni correction revealed that seizure frequency at 4, 8, and 12 weeks (post-W4, -W8, -W12, respectively) of treatment was significantly lower than during the baseline period (*p* < 0.0001). The points on the violin plot represent the color difference values between time points, while the red dot represents the median.

Seizure frequency was assessed during a 12-week pre-treatment baseline period and a 12-week CBD treatment period, with assessments performed every 4 weeks. A notable decrease in seizure frequency was observed after CBD administration. The Friedman test confirmed a statistically significant difference in seizure frequency across pre-treatment and post-treatment time points (χ^2^ = 25.27, *p* < 0.0001; Table 1; Figure 1).

**Figure 2:**
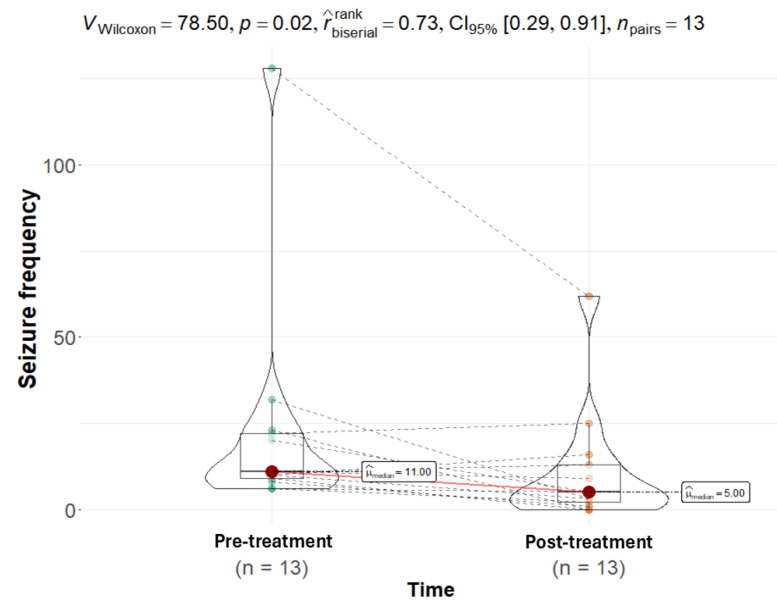
A Wilcoxon matched-pairs signed-rank test demonstrated a statistically significant change in seizure frequency over 12 weeks, with data before and after the CBD intervention (*p* = 0.02). The median seizure frequency post-intervention (median = 5) was lower than pre-treatment (median = 11). The points on the violin plot represent the color difference values between pre- and post-treatment, while the red dot represents the median. The pink line explicitly links the median of the pre-treatment distribution to the post-treatment distribution, while the dashed line links corresponding individual data points.

The pre-treatment baseline revealed considerable variability, with a median seizure frequency of 11 (IQR 9–22), consistent with the heterogeneous nature of refractory epilepsy. Throughout the 12-week treatment period, the median seizure frequency for the entire cohort decreased to five (IQR 2–13). This overall reduction was further supported by Wilcoxon signed-rank test results for the entire cohort (*p* = 0.02; Figure 2).

Subgroup analysis indicated that the overall effect was driven by a strong, albeit non-significant, trend in the idiopathic epilepsy (IE) subgroup (*p* = 0.066). In contrast, no evidence of treatment effect was observed in the structural epilepsy (SE) subgroup (*p* = 0.375).

*Responder and Seizure-Free Analysis*

Applying a ≥50% reduction in seizure frequency as the responder criterion, eight of the 13 dogs (61.5%) achieved responder status. Notably, two dogs with IE were seizure-free throughout the 12-week treatment period, and two additional dogs with IE experienced seizure-free periods every 4 weeks. Two dogs with SE remained seizure-free for eight consecutive weeks.

*Seizure Cluster Outcomes*

**Figure 3:**
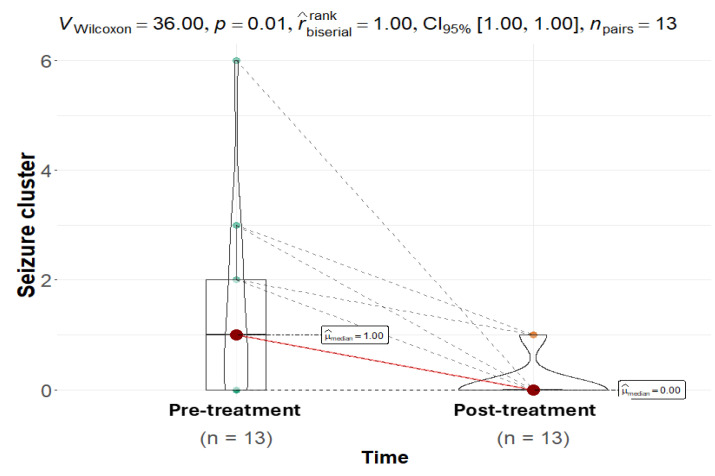
A Wilcoxon matched-pairs signed-rank test revealed a statistically significant difference in seizure clusters before and after the administration of CBD (*p* = 0.01). The median number of seizure episodes per 12-week period post-intervention (median = 0) was notably lower compared to the pre-treatment phase (median = 1). The points on the violin plot represent the color difference values between pre- and post-treatment, while the red dot represents the median. The pink line explicitly links the median of the pre-treatment distribution to the post-treatment distribution, while the dashed line links corresponding individual data points.

**Figure 4:**
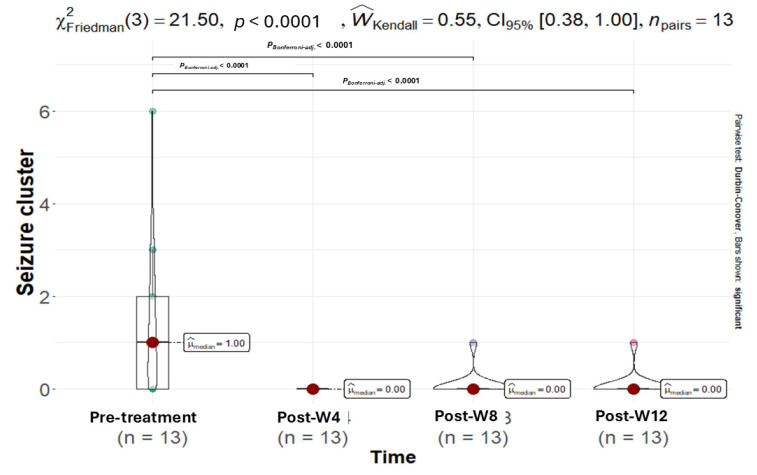
A Friedman test indicated a statistically significant reduction in seizure clusters over time (χ^2^ = 21.50, *p* < 0.0001). Post hoc analysis with Bonferroni correction confirmed seizure clusters were significantly lower at all post-treatment intervals—4, 8, and 12 weeks (median = 0) compared to the pre-treatment phase (median = 1) (*p* < 0.0001). The points on the violin plot represent the color difference values between time points, while the red dot represents the median.

The median number of seizure clusters decreased to zero for the entire cohort and remained at zero throughout the 12-week follow-up period (Figure 3). The Friedman test revealed a highly significant reduction in clusters in the whole cohort (p < 0.0001), as well as in both the SE (*p* = 0.0163) and IE (*p* = 0.0102) subgroups (Table 1, Figure 4). However, a direct pre-post comparison using the Wilcoxon signed-rank test showed only a strong trend in the IE subgroup (*p* = 0.057) and a non-significant change in the SE subgroup (*p* = 0.174), suggesting that the Friedman test captured a substantial temporal effect driven by a sustained decrease.

### 3.3. Clinical Evaluation of Safety Profiles and Quality of Life

The safety profile was evaluated using clinical assessments and hematological/biochemical parameters during the 12-week intervention period (Table 2). Body weight remained stable in most dogs with minor fluctuations (within ±0.5 kg). Hematological and serum biochemical parameters showed significant patterns across the baseline and post-treatment periods, with stability in most hematological and renal parameters. Individual variability was noted, with eosinophilia in 38% of the dogs. Most hematological and biochemical parameters remained within reference ranges, with no statistically significant changes over the 12 weeks, indicating physiological stability despite the addition of CBD.

The data demonstrated the individualized and response-driven nature of the “start-low, go-slow” protocol. Only three dogs achieved a maximum target dose of 2.5 mg/kg. While two-thirds of the dogs were titrated to a moderate or high dose (≥1.5 mg/kg), one-third remained on a very low dose (0.5–0.75 mg/kg) throughout the 12 weeks.

### 3.4. Owner-Perceived Outcomes and Adverse Events

Owner-perceived outcomes regarding seizure control and adverse events were assessed using an 11-item Likert scale and a seizure diary (n = 12 owners responded to the survey). A majority of owners (10/12) perceived at least a “moderately decreased” seizure frequency, with a strong finding that 8 of the 12 owners reported a “much” or “markedly decreased” frequency. Only 2 participants reported a “minimally decreased” frequency. The owner’s perception of the response to seizure severity was similarly positive: 8 owners reported a “markedly decreased” severity, 3 reported a “much decreased” severity, and 1 reported a “minimally decreased” severity.

Adverse events were generally infrequent and documented as rare or very rare (Likert scale scores 1 or 2). The reported events included agitation or restlessness, diarrhea or loose stools, urinary incontinence, and hypersalivation. Importantly, vomiting was not reported in this cohort. Conversely, 10 of the 12 owners reported positive behavioral indicators, specifically increased appetite and improved Bright-Alert-Response and vitality, with moderate-to-frequent occurrences. Overall, 10 owners (83.3%) reported an improvement in the quality of life for both them and their dogs while maintaining a positive attitude towards the increased costs associated with CBD use. All summarized data from owners’ observations are available in Supplementary Table S2.

## 4. Discussion

This prospective pilot study provides valuable insights into the potential benefits and safety of CBD as adjunctive therapy in the context of canine refractory epilepsy. Our findings indicate that the co-administration of CBD with conventional AEDs significantly reduced seizure frequency and cluster events in the entire cohort, albeit with notable inter-individual variability. These results are consistent with emerging evidence supporting the anticonvulsant properties of CBD and underscore critical safety considerations for clinical translation.

*Potential Efficacy in Refractory Epilepsy*

The observed ≥50% seizure reduction in this pilot study generally aligns with findings from previous veterinary investigations of CBD’s anticonvulsant effects. While a double-blind crossover study reported a 24.1% reduction in seizure days at a higher CBD dose (9 mg/kg/day) [15], other studies have yielded mixed results. For instance, a 2019 randomized controlled trial using a lower dose (2.5 mg/kg twice daily) reported a 33% median reduction in seizure frequency. Still, it did not demonstrate a significant difference in the proportion of dogs achieving ≥50% seizure reduction compared with placebo [13]. More recently, a study exploring a combination of CBD and CBDA (2 mg/kg twice daily) observed a mean seizure frequency reduction from 8.0 to 5.0, with 43% of dogs achieving ≥50% seizure reduction, a higher proportion than that reported in a 2019 trial [14].

These findings suggest that CBD may counteract pharmacoresistance through multiple mechanisms [13,20]. The antagonistic effects of CBD on G-protein-coupled orphan receptors (GPR18 and GPR55), and on vanilloid receptors (TRPA1 and TRPV1-4) are noteworthy [20,21]. Additional pharmacological targets, including partial agonism at 5-HT1A receptors and reduced production of pro-inflammatory cytokines through its agonistic effect on peroxisome proliferator-activated receptor-gamma (PPARγ), may further contribute to its efficacy in refractory epilepsy, as supported by ex vivo and clinical observations in treatment-resistant patients [11,21,22].

The response patterns in our study varied, with some dogs exhibiting rapid improvement and others showing gradual or fluctuating reductions—notably, a bimodal response, with some dogs becoming nearly seizure-free. In contrast, others derived minimal benefit, mirroring findings in human trials of CBD for treatment-resistant epilepsy [8], implying its potential efficacy in a distinct subpopulation. This heterogeneity may stem from genetic differences in cannabinoid receptor expression or epilepsy etiology, warranting further pharmacogenomic studies. Additionally, the weak correlation between pre- and post-treatment outcomes underscores substantial inter-individual variability, consistent with the bimodal response. Longitudinal assessments collectively suggest that while CBD holds promise for refractory epilepsy, its efficacy varies significantly across individuals.

The evidence of a differential response indicated that the temporal patterns of seizure reduction were non-uniform, with specific time points demonstrating greater efficacy than others. This variability emphasizes the critical role of longitudinal monitoring in evaluating the response to CBD treatment, as some animals require extended periods to exhibit therapeutic benefits. In contrast, others experience either no improvement or only immediate improvement. These divergent response trajectories suggest the potential presence of distinct pathophysiological subtypes of refractory canine epilepsy, which may necessitate tailored therapeutic approaches.

The pronounced reduction in seizure clusters represents a clinically significant outcome, as clusters are associated with poor prognosis and therapeutic challenges [5]. CBD’s potential interruption of ictogenesis via GABAergic enhancement and glutamate modulation may explain this effect, offering a promising strategy for cluster management where conventional therapies often fail [23].

*Safety, Tolerability Profile, and Quality of Life*

The analysis confirmed that the majority of hematological parameters did not show statistically significant changes over the 12-week intervention period. Similarly, most biochemical parameters remained stable. Hematological and renal parameters remained stable throughout the study, consistent with CBD’s established safety profile in dogs [13,14,18,24]. This indicated a general absence of widespread adverse effects on erythropoiesis, coagulation, or renal function attributable to CBD co-treatment during the observation period. Notably, a large proportion of the cohort (12 of 13 dogs) exhibited elevated eosinophil levels at baseline, a condition that CBD co-administration did not statistically exacerbate. Eosinophilia is a rare adverse effect of phenobarbital administration in dogs. This condition may be present as part of a broader syndrome, such as Drug Rash with Eosinophilia and Systemic Symptoms (DRESS), or as a more localized reaction [25]. Additionally, certain dogs may exhibit hypersensitivity to AEDs, resulting in adverse reactions, specifically Anticonvulsant Hypersensitivity Syndrome (AHS) [26].

However, the statistically significant elevation in ALP levels across all post-treatment intervals raises concerns about hepatic stress. This aligns with previous studies conducted on dogs [13,14,15]. Furthermore, this is corroborated by human research in which CBD-induced ALP elevation (without concurrent bilirubin increase) was attributed to enzyme induction rather than hepatocellular damage [27,28].

Although the observed abnormal baseline and elevated ALT levels in certain dogs, particularly during the initial four weeks, did not reach statistical significance, they may suggest hepatotoxic interactions between CBD and conventional AEDs. CBD is known to inhibit cytochrome P450 enzymes, notably CYP3A4 and CYP2C19 [29,30], thereby potentially impairing phenobarbital metabolism. This issue is further complicated by polypharmacy in this group, with 2–6 AEDs administered simultaneously. Each dog was treated with phenobarbital, a CYP450 enzyme inducer, in addition to other AEDs. This regimen may increase metabolic stress caused by CBD, paralleling the interaction between CBD and valproate observed in human studies. Mechanisms such as mitochondrial dysfunction, enzyme inhibition, and transporter effects are plausible across species, as documented by Beers et al. [31].

In this context, the patterns observed in our study likely indicate sustained hepatic stress, potentially reflecting pre-existing AED-induced enzyme induction, which CBD did not ameliorate. Concurrent ALT elevations noted in several dogs suggest a degree of hepatocellular injury, thus warranting vigilant monitoring of potential hepatotoxicity. Nonetheless, these findings align with those of earlier studies, which also reported variations in hepatic enzyme markers in dogs following CBD administration [32,33].

Collectively, these findings showed that CBD co-treatment did not cause broad hematological or renal disruption. However, this may be associated with sustained elevation of hepatic enzymes in most dogs, consistent with AED-related metabolic stress. These warrant the monitoring of liver function in at-risk patients. The significant inter-individual variability in the pharmacokinetic pattern of plasma CBD underscores the need for individualized monitoring and rigorous evaluation of CBD safety in epileptic canines [18]. Subsequent analyses should prioritize longitudinal assessment of hepatobiliary markers, trends in eosinophilia, and correlations with CBD/AED dosing.

The use of CBD products in dogs is typically well tolerated, with none to moderate adverse effects [10,24,34]. Reported side effects include reduced appetite, vomiting, and variations in stool consistency, such as soft stools or diarrhea [14,15]. More serious concerns involve potential elevations in liver enzyme levels, particularly when CBD oil is used concomitantly with other anti-seizure medications, or in patients at increased risk [28].

Pet owner surveys consistently show a positive perception of CBD use in animals [35,36]. Our survey reinforced this, with all participants expressing a favorable view of giving CBD to their pets. Similarly, earlier publications related to cannabis product use in pets indicated high owner satisfaction, whether for therapeutic reasons or as a supplement [36,37].

Interestingly, our findings suggest that owners perceive a greater drop in seizure frequency with CBD than in earlier studies [14]. Although episodes of loss of balance were reported in some instances in our research, owners consistently documented that their dogs remained alert and vital, with increased appetite. The participating owners also reported other adverse events, including lethargy, urination, and thirst. These observations corroborate those reported in previous studies [14,32]. In this regard, variable systemic drug exposure, driven by factors such as dosage, treatment duration, formulation, and interactions with concurrent medications, can significantly influence adverse event profiles observed with CBD [13,17].

Nonetheless, owners of the participating dogs expressed a positive to very positive attitude regarding the improved quality of life experienced by both their pets and them after CBD co-treatment. Given this favorable context, owners showed moderate to high acceptance of the increased costs associated with CBD use. This finding aligns with previous reports indicating that owners seek affordable pricing [36].

However, it is worth noting that direct comparisons of owner perception and canine QoL in dogs with idiopathic epilepsy are challenging. Each study’s unique design for evaluating these aspects makes cross-investigation comparisons difficult [4]. Our findings are consistent with those of Bride [38], who reported that geographic, cultural, and socioeconomic factors may influence the diagnosis and treatment of epilepsy. These disparities may ultimately impact both QoL measures and owner perspectives.

*Clinical Implications and Limitations*

It is essential to acknowledge the limitations inherent in the design, execution, and clinical implications of this study. This single-arm, quasi-experimental pretest–post-test design was a pragmatic choice for a pilot investigation in a real-world setting, particularly for a refractory, inherently difficult-to-study population. Although practical, this study has inherent limitations that affect the strength of its conclusions. The relatively small cohort size, comparable to that of prior canine CBD trials [13,14], reduces statistical power to detect subgroup differences. This reflects the challenges of recruiting refractory epilepsy cases within a constrained timeframe, as many owners declined participation due to financial constraints or prior treatment fatigue.

Our pragmatic “start-low, go-slow” titration strategy (0.5 → 2.5 mg/kg BID) was designed to minimize severe adverse events while maintaining efficacy—a key consideration given owner concerns regarding CBD-related side effects. These findings challenge the one-dose-fits-all paradigm, which advocates personalized titration of CBD in veterinary neurology. This aligns with Bradley [24], who noted that customizing CBD dosages for each dog could lead to more comparable plasma concentrations, potentially improving effectiveness. However, the cautious dosing approach used in our study, which capped the maximum dose at 2.5 mg/kg, may have inadvertently restricted the potential therapeutic benefits if higher doses were required to achieve optimal seizure control. Consequently, the observed outcomes may reflect sub-therapeutic dosing in some cases, highlighting the balance between tolerability and efficacy.

Although numerous studies on canine epilepsy and the use of CBD for this specific disease have been conducted in Europe and North America [10,38], to our knowledge, this study represents the first scientific report originating from Asia to investigate cannabidiol as a potential adjunctive therapeutic intervention for this neurological disorder in dogs with epilepsy.

## 5. Conclusions

This study supports the potential use of CBD as adjunctive therapy for refractory canine epilepsy, particularly in cluster-prone individuals. However, increases in hepatic enzyme activity underscore the necessity for rigorous hepatic monitoring, especially in dogs receiving hepatically metabolized AEDs and those presenting with baseline hepatic enzyme elevations. Future research should focus on individualized dosing regimens and pharmacovigilance protocols to maximize safety in this vulnerable population.

## Figures and Tables

**Table 1:** Median (IQR) seizure frequency and cluster of 13 dogs. A Friedman test was conducted to compare seizure frequency and seizure clusters across time points (pre-intervention, for 12 weeks, and post-treatment at 4, 8, and 12 weeks). IE = Idiopathic epilepsy, SE = Structural epilepsy.

	Time Point (Week)				*p*-Value
Dog (n)	Pre-Intervention/	Post-Intervention/Median (IQR)			
	Median (IQR)	Post-Week-4	Post-Week-8	Post-Week-12	
Seizure frequency					
SE (4)	10(8–21)	1.5(0.75–2.25)	0.5(0–1.0)	4(0.75–5.25)	0.0403
IE (9)	11(9–22)	1(0–5)	5(0–9)	0(0–3)	0.0042
All (13)	11(9–22)	1(0–3)	1(0–5)	1(0–3)	<0.0001
Seizure cluster					
SE (4)	1(0.5–3.5)	0(0)	0(0)	0(0)	0.0163
IE (9)	1(0–2)	0(0)	0(0)	0(0)	0.0102
All (13)	1(0–2)	0(0)	0(0)	0(0)	<0.0001

**Table 2:** Comparison of blood parameters between baseline and—4-, 8-, and 12-week post-intervention. Data were analyzed using repeated-measures ANOVA with Dunnett’s test or Friedman test with Dunn’s test, as appropriate. *p* < 0.05 vs. baseline. Parameters marked with an asterisk were non-parametric and are displayed as median (range).

Parameter (Abbreviation)	Normal Range (Unit)	Baseline		Week-4		Week-8		Week-12		*p* Value
		Mean	SD	Mean	SD	Mean	SD	Mean	SD	
Packed cell volume (PCV)	35–57%	42.39	5.45	42.72	5.69	43.45	5.86	42.98	5.86	0.829
Red blood cell (RBC)	4.95–7.87 × 10^6^/μL	6.31	0.91	6.22	0.99	6.16	0.96	6.31	1.17	0.773
Hemoglobin (HGB)	11.9–18.9 g/dL	15.27	1.51	15.22	2.14	15.03	1.44	15.55	2.07	0.677
White blood cell (WBC)	5–14.1 × 10^3^/μL	12.52	4.00	12.37	4.69	12.02	4.00	11.26	4.57	0.227
Platelet count (PLT)	211–621 × 10^3^/μL	406.92	150.51	382.00	148.62	380.00	151.11	393.85	153.84	0.712
Neutrophils (NEU)	58–85%	68.77	7.78	70.18	8.32	69.05	8.73	67.56	7.96	0.539
Lymphocytes (LYMP)	8–21%	19.77	6.30	17.65	6.00	18.79	7.32	18.90	6.44	0.457
Monocytes (MONO)	2–10%	4.44	2.29	4.89	2.64	5.25	0.85	5.18	0.91	0.571
Eosinophils (EO)	0–9%	6.94	5.71	7.21	4.23	6.68	4.40	8.09	4.98	0.486
Basophils (BASO) *	0–1%	0.1 (0–0.2)		0.1 (0–0.6)		0.1 (0–0.4)		0.1 (0–0.3)		0.615
Alanine aminotransferase *	10–109 U/L	63 (44–152)		84 (50–503)		70 (34–370)		82 (48–324)		0.058
Alkaline phosphatase *	8–76 U/L	727 (294–1314)		944 (354–5027)		976 (394–6752)		1156 (408–7605)		0.001
Aspartate aminotransferase *	15–56 U/L	28 (17–54)		28 (16–82)		27 (16–75)		30 (18–58)		0.970
Blood urea nitrogen (BUN)	8–28 mg%	16.46	5.72	15.00	4.24	15.31	4.46	15.46	6.31	0.607
Creatinine (CRE)	0.50–1.70 mg%	0.79	0.23	0.84	0.23	0.79	0.27	0.84	0.26	0.483
Total protein (TP)	5.4–7.5 g/dL	5.97	0.38	6.15	0.53	5.80	0.57	5.86	0.63	0.202
Albumin (ALB)	2.3–3.1 g/dL	3.01	0.37	2.95	0.44	2.86	0.47	2.86	0.45	0.141
Blood glucose (BG)	80–120 mg/dL	112.92	29.89	108.00	25.06	117.15	32.33	117.62	32.12	0.360

## Data Availability

Data is contained within the article.

## Supplementary Materials

The following supporting information can be downloaded at: https://www.mdpi.com/article/10.3390/ani15243614/s1. Table S1. The demographic and medications data; Table S2. Questionnaire Adverse Event and perception use of CBD product; Table S3. Seizure Calendar.

## Abbreviations

AEDs	Anti-epileptic drugs
ALP	Alkaline Phosphatase
ALT	Alanine aminotransferase
CBD	Cannabidiol
IE	Idiopathic epilepsy
QoL	Quality of life
SE	Structural epilepsy
