# Supplementary material for: An Exploratory Study of Cannabidiol as an Adjunctive Treatment for Refractory Epilepsy in Dogs
Source: Animals (Basel). 2025 Dec 15;15(24):3614. doi: 10.3390/ani15243614 (PMC12729276; doi:10.3390/ani15243614)
Supplement: Supplementary file 1 [file animals-15-03614-s001.zip › animals-4013558-supplementary.pdf]

Supplementary Table (S1).

The demographic and medications data

Dog	Breed	Sex		Age		Body weight (Kg)		Seizure type	AEDs administration						
(Number)		Sex	Status	At onset	At study	Week-0	Week-12		Phenobarbital	Zonisamide	Levetiracetam	Potassium bromide	Topiramate	Gabapentin	CBD max-dose
1	Chihuahua	Female	Intact	1 Y	2 Y	3.5	2.8	SE	Yes	Yes	Yes	Yes	None	Yes	1.5
2*	Chihuahua	Male	Intact	5 Y	10 Y	7.8	6.7	IE	Yes	Yes	Yes	Yes	None	Yes	2
3	Chihuahua	Male	Intact	6 Y	7 Y	3.7	3.95	SE	Yes	None	Yes	Yes	None	None	2
4	Chihuahua	Male	Neutered	3 Y	4 Y	3.4	3.25	SE	Yes	None	Yes	Yes	None	None	2.5
5*	Chihuahua	Female	Neutered	5 mth	3 Y	3.85	4.3	IE	Yes	Yes	Yes	Yes	Yes	Yes	2.5
6*	Pomeranian	Male	Intact	5 mth	3 Y	5.55	4.95	IE	Yes	Yes	Yes	None	None	Yes	2
7*	Labrador retriever	Male	Intact	1 Y	6 Y	43	43.2	IE	Yes	None	Yes	None	None	Yes	2
8*	Siberian husky	Male	Intact	2 Y	5 Y	30.95	27.85	IE	Yes	None	Yes	None	None	Yes	0.5
9	Beagle	Male	Neutered	2 Y	3 Y	15.4	15	IE	Yes	None	None	Yes	None	None	0.5
10	Siberian husky	Male	Intact	1 Y	1.5 Y	24.35	27.6	IE	Yes	Yes	None	Yes	None	Yes	2
11	Siberian husky	Male	Neutered	3 Y	7 Y	49.25	50.2	IE	Yes	Yes	Yes	None	None	Yes	2.5
12	Chihuahua	Female	Intact	8 Y	8 Y	3.45	3.3	SE	Yes	Yes	Yes	None	None	Yes	0.75
13	Crossbreed	Male	Neutered	1 Y	4 Y	6.9	7.35	IE	Yes	None	Yes	Yes	None	Yes	0.5

Remarked: Dog code number with asterisk indicated complete IVETF level-II. Other IE dogs lack bile acid and MRI testing.

CBD max-dose (mg/kg) means the maximum dose of CBD at 12-week of study while all dogs start at 0.5 mg/kg (baseline)

Supplementary Table (S2). Questionnaire Adverse Event and perception use of CBD product

**Topic / Observation**	**Scoring Level (Specify a number from 1 to 5)**:<br>1 = Not observed and very rarely, 2 = Rarely observed,<br>3 = Moderately observed, 4 = Frequently observed,<br>5 = Most frequently observed				
	**5**	**4**	**3**	**2**	**1**
1. Ataxia/loss of balance/disorientation					
2. Agitation or restlessness					
3. Lethargy and weakness					
4. Polyphagia; increased appetite					
5. Bright-Alert-Response and vitality					
6. **Vomiting**					
7. Diarrhea or loose stools					
8. **Urinary incontinence or pollakiuria**					
9. **Xerostomia; dry mouth / Polydipsia; increased thirst**					
10. Hypersalivation or ptyalism					

Severity Scale (Treatment Response Assessment):

5 = Markedly decreased, 4 = Much decreased, 3 = Moderately decreased, 2 = Minimally decreased, 1= No decrease					
	5	4	3	2	1
1. Owner's perception of the response to seizure frequency					
2. Owner's perception of the response to seizure severity					
5 = Very much improved, 4 = Much improved, 3 = Moderately improved, 2 = Minimally improved, 1 = No improved					

	5	4	3	2	1
3. Owner's attitude toward the dog's quality of life					
4. Owner's attitude toward their own quality of life					
5 = Most acceptable, 4 = Much acceptable, 3 = Moderately acceptable, 2 = Minimally acceptable, 1 = Not acceptable					
	5	4	3	2	1
5. Owner's attitude toward the increased cost associated with CBD use					
6. Behavioral changes; positive or negative outcome – please describe					
7. Other aspects related to the quality of life of the dog and its owner, including attitudes or perceived responses to CBD; please describe					

------------------- Thank you for your kind cooperation ----------------

Summarized data from the owner 'observation (n)

**Topic / Observation**	**Scoring Level (Specify a number from 1 to 5)**: 1 = Not observed and very rarely, 2 = Rarely observed, 3 = Moderately observed, 4 = Frequently observed, 5 = Most frequently observed				
	**5**	**4**	**3**	**2**	**1**
1. Ataxia/loss on balance/disorientation			2	2	8
2. Agitation or restlessness				5	7
3. Lethargy and weakness		1	3	3	5
4. Polyphagia; increased appetite		3	4	3	2
5. Bright-Alert-Response and vitality		3	5	2	2
6. Vomiting					12

7. Diarrhea or loose stools				2	10
8. Urinary incontinence or pollakiuria				3	9
9. Xerostomia; dry mouth / Polydipsia; increased thirst			3	5	4
10. Hypersalivation or ptyalism				2	10

Severity Scale (Treatment Response Assessment):

5 = Markedly decreased, 4 = Much decreased, 3 = Moderately decreased, 2 = Minimally decreased, 1= No decrease					
	**5**	**4**	**3**	**2**	**1**
1. Owner's perception of the response to seizure frequency		5	3	2	2
2. Owner's perception of the response to seizure severity		8	3	1	
5 = Very much improved, 4 = Much improved, 3 = Moderately improved, 2 = Minimally improved, 1 = No improved					
	**5**	**4**	**3**	**2**	**1**
3. Owner's attitude toward the dog's quality of life	4	6	1	1	
4. Owner's attitude toward their own quality of life	6	4	1	1	
5 = Most acceptable, 4 = Much acceptable, 3 = Moderately acceptable, 2 = Minimally acceptable, 1 = Not acceptable					
	**5**	**4**	**3**	**2**	**1**
5. Owner's attitude toward the increased cost associated with CBD use	2	4	5	1	

Supplementary Table (S3). Seizure Calendar

Seizure Calendar for: …………………………………….. Pet ID…………………………………Year…………………….………………..

Date	January	February	March	April	May	June	July	August	September	October	November	December
1												
2												
3												
4												
5												
6												
7												
8												
9												
10												
11												
12												
13												
14												
15												
16												
17												
18												
19												
20												
21												
22												
23												
24												
25												
26												
27												
28												
29												
30												
31												
Total												

**Should the animal display any of the following symptoms —seizure activity persisting for more than 5 minutes or experiencing more than two seizure episodes within a single day—it is crucial to seek immediate veterinary attention, as the pet may be undergoing status epilepticus.*

*** Any additional questions or concerns should be addressed through direct contact.*
